# Supplementary material for: A longitudinal analysis of selective motor control during gait in individuals with cerebral palsy and the relation to gait deviations
Source: PLoS One. 2023 Jul 31;18(7):e0289124. doi: 10.1371/journal.pone.0289124 (PMC10389713; doi:10.1371/journal.pone.0289124)
Supplement: Attachment — Submitted filename: Editor and reviewers comments 250423.docx [file pone.0289124.s001.docx]

**Dear editor and reviewers,**

Thank you for taking the time to scrutinize the manuscript, and providing feedback on where and how it can be improved.

Our responses to your comments are written below in italic, whilst a yellow highlight has been used to represent the changes made within the manuscript (pages and lines are referred the revised manuscript with track changes).

**Editor Comments:**

1. The manuscript presents interesting results. However, there are some methodological concerns that could have a negative impact on the results:
- why have the authors choose only 16 typical developed children for control reference values? How was this sample size established? They should be at least age-matched. There is a difference between the age range in CP participants and TD ones. What about these children characteristics - height, weight?

*Thank you for highlighting this oversight.* *The TD group used in this manuscript was a convenience sample, in order to provide a reference for calculating the gait profile score and walk-DMC values. As such, the characteristics of this cohort were omitted. This has been rectified in* table 1. *With respect to patient characteristics, these parameters have been shown to be stable between the age of 4-18 (10.1111/dmcn.12826 and 10.1016/j.gaitpost.2018.06.129). As such we do not consider the heterogeneous characteristics of the TD group to have influenced the results. These explanations have been added to the revised manuscript:*

*Page 6, Line 23- page 7 line 4:* *In addition, a convenience sample of 16 TD children were included to provide control reference values (Table 1). The main outcome parameters of this study have been shown to be stable between the age of 4-18 (12,22). As such we do not consider the sample size of heterogeneous characteristics of the TD group to have influenced the results.*

2. walking barefoot on the treadmill was comfortable for children? Could this have affected the gait analysis?

*Thank you for your comment. In over 700 clinical gait analyses, there have been no* *complaints of discomfort when walking barefoot on the instrumented treadmill. We therefore do not consider this to have influenced the analysis. This has been added to the manuscript*

*Page 10, Line 2-3: No adverse events were reported during the assessments and none of the participants complained of discomfort when walking barefoot on the instrumented treadmill.*

3. In the results section (first paragraph) it is mentioned that in both assessments most participants were regular users of ankle-foot orthoses. Were they wearing the orthoses during gait assessments?

*This should have been clearer in the manuscript, and has been rectified:*

*Page 9, Line 8-10: Individuals that are regular users of ankle-foot orthoses were usually assessed both barefoot and with their orthoses. However, for this study, only data from the barefoot condition were included.*

4. what about the difference between CP participants that used handrails for support while walking and those who did not?

*Thank you for your comment. It was found that* *most participants (n=19) did not use handrails. However, those who did use handrails were GMFCS levels II-III, indicating a lower functional level. Despite this, the initial analysis did not reveal any significant influence on the results. It should also be noted that these participants used handrails in both assessments, and this has been clarified in the manuscript as follows:*

*.*

*Page 9, Line 24-25: Both the first and second assessment protocols were identical, including the use of handrails by those participants who needed to do so.*

*Page 14, Line 14-16: Most participants (n=19) did not use handrails during the assessments; an initial analysis did not reveal any significant influence of using handrails on the results.*

*In addition to the requested clarifications/changes, efforts have been made to improve the grammar and fluidity of the manuscript.*

Reviewer's Responses to Questions

**Comments to the Author**

Reviewer #1: Dear Authors, thank you very much for submitting your manuscript to PLOS ONE. The readers of the PLOS ONE will be interested in the topic of present study. I have some concerns about your study. I indicated my comments for each section of the manuscript.

*Thank you for the positive feedback. We will try to the best of our ability to address your concerns.*

*In addition to the requested clarifications/changes, efforts have been made to improve the grammar and fluidity of the manuscript.*

Abstract
Keywords
1. Mesh terms do not match the developmental delay and muscle synergies. Please double-check your keywords using MeSH.

*Thank you for the comment. We updated the keywords using MeSH:*

*Cerebral Palsy, Gait, Electromyography, Biomechanics, Kinematics, Developmental delay disorder*

Introduction
2. Please update the authors name in the reference number one. (eg: Damiano, DiL not correct)

*Thank you for noticing this. The reference has been corrected:*

*Graham HK, Rosenbaum P, Paneth N, Dan B, Lin JP, Damiano DL, et al. Cerebral palsy. Nat Rev Dis Prim. 2016;2.*

- Introduction is well written. The authors used current references from the literature.

Method
3. Assessments and sample size were described in detailed. But authors should indicate the calculation of sample size. How did you decide to involve 16 health peers into the study?

*Thank you for highlighting this oversight. This has been addressed in the text.*

*Page 6, Line 23- page 7 line 4: In addition, a convenience sample of 16 TD children were included to provide control reference values (Table 1). The main outcome parameters of this study have been shown to be stable between the age of 4-18 (12,22). As such we do not consider the sample size of heterogeneous characteristics of the TD group to have influenced the results.*

Results
4. You should present the sociodemographic characteristics of children without CP (typically developing peers) such as age, gender, body mass etc.

*This has been added to table 1.*

5. Are there any adverse events in the groups during the assessments?

*No adverse events were seen in the groups during the assessments. This was added to the manuscript:*

*Page 10, Line 2-3: No adverse events were reported during the assessments and none of the participants complained of discomfort when walking barefoot on the instrumented treadmill.*

6. You should include the effect sizes for differences within group differences in Table 2.

*We added the effect sizes for within group differences in Table 2.*

Discussion
7. Limitations are more comprehensive. I think sample size is one of the limitations of the study.

*Thank you for your comment. We have expanded upon this in the limitation section.*

*Page 23, Line 14-16: Regarding the limitations of this study,* *it should be acknowledged that the sample size, although sufficiently powered, is modest. Therefore, while the findings of this study may be valuable and informative, they should be interpreted with caution*

8. Please also include the strengths of the study. For example, using objective evaluation methods are one of the strengths of the study.

*Thank you for bringing this to our attention. This has been added.*

*Page 24, Lines 9-18:* *This study has several strengths that enhance the validity and significance of its findings. Firstly, it incorporates an objective evaluation of muscle activity and gait kinematics. Second, a minimum of 30 strides per leg were included for most participants. This method not only strengthens the validity of our study results but also enhances the reliability of the data collected. Additionally, to the best of our knowledge, this study is the first to evaluate longitudinal changes in SMCg in a group of individuals with CP without any surgical interventions between assessments (including botulinum toxin injections). It also examines whether changes in SMCg are associated with variations in gait deviations. As a result, this study provides valuable insights into the natural progression of SMCg during gait, previously unreported.*

Reviewer #2: General comments
This is an interesting study. The sbmitted manuscript is generally well thought-through and adresses relevant and novel topics. However, I have some major concerns and I would advice against publication of the study in it’s current form.

My concerns are mainly about the interpretation and conclusions that the authors draw from the study results. I believe that more nuance would be in place and too much emphasis is put on some minor results.
Already in the abstract the authors claim quite strongly that ‘individuals with CP demonstrated longitudinal changes in SMCg when evaluated on an individual level’ and ‘add to the evidence that SMCg in individuals with CP may continue to change over time’. Unfortunately, this is not supported by a statistical change in SMCg in the results. Although I am generally in favor of reporting individual results in addition to group statistics, especially in a heterogeneous population like individuals with CP, the authors frame it as an improvement on individual level. While in fact, the only valid conclusion would be that they found no change in SMCg. Of course, further exploration into the reasons for the absence of any significant change is valuable, but the way it is presented now is confusing and in some sections even misleading.

Secondly, while the authors put a lot of emphasis on the outcomes of the hip joint, this data is not presented clearly in the results. Presentation of data supporting their conclusions is missing (see specific comments below). Moreover, I have some methodological concerns on the outcomes of the hip joint which are listed below in detail.
My final general concern is about the discussion paragraphs. This section should be revised thoroughly as in it’s current form, it lacks a critical evaluation of the findings and implications of the methodological choices made by the authors.

*Thank you for your frank appraisal. Using your feedback, we have improved the manuscript.*

*We made relevant changes in the manuscript to address your concerns.*

Abstract:

*The conclusion in the abstract was rephrased:*

*Page 2, Line 18-23:* *On average, there were no significant longitudinal changes in SMCg. However, there was considerable variability between individuals, which may relate to changes in hip joint kinematics. This suggests that a combination of neural capacity and biomechanical factors influence lower limb muscle co-activation in individuals with CP, with a potential important role for the hip muscles. These findings highlight the importance of taking an individualized approach when evaluating SMCg in individuals with CP.*

Introduction
Page 4 – line 4: Hypotheses on significant changes in SMCg between assessments versus changes on an individual level. It is unclear what the authors mean by ‘changes on an individual level’. Assuming that a statistical design is used to test within-subject changes over time, doesn’t that represent individual changes? Please rephrase.
*Thank you for your comment. We rephrased it.*

*Page 5, Line 22- page 6 line 2:* The first hypothesis is that despite an expected high variability in SMCg (6,16,21), there will be no significant change in this measure between assessments. The second hypothesis states that there will be significant relationships between changes in measures of SMCg and gait deviations (11).

Materials and methods
1. Participants
Page 5 – line 8: The age range of 4-18 years is quite large. Especially in combination with the time range between assessments (results, page 10: 193-992 days). How was this distributed over the group? I.e. did younger children have more/less days in-between assessments?

*The time between assessments was largely variable between ages. The Pearson correlation between age to the time between assessment is very low (r_p_=-0.012, p=0.957). This has been added to the text and been clarified in the manuscript.*

*Page 14, Line 11-12: There was no significant relationship between the interim period and age, whether the individuals with CP were younger or older.*

*And- Page 19, Lines 1-8: Prior to evaluating changes in SMCg over time, we explored the relationship between age and walk-DMC at the time of the first assessment, without any significant findings. This is in line with a previous study in a very large cohort (12). Additionally, Cappellini et al also demonstrated very limited age-related changes in muscle synergies in children with CP, based on a single assessment in a wide age range of participants (13). It has also been shown that SMCg in individuals with CP can often resemble the synergies observed in much younger TD children, highlighting not only the delay but also stagnation in SMCg development (40).*

2. Could the authors also provide information on the reason why children were seen for gait analysis?
*Of course. This has been clarified in the manuscript.*

*Page 6, Line 12-15:* *Individuals were referred for a clinical gait analysis to* *determine the benefit of monitoring gait function to improve: 1) orthopedic management, 2) spasticity management, 3) effect of ankle foot orthoses, 4) assistive devices and 5) physiotherapy treatment.*

Data collection
1. Page 5 – line 24: How was GMFCS scored? Did the authors expect this outcome to change, as it is used as an outcome for function?

*The GMFCS level was* *scored by the physiotherapist conducting the gait analysis, and was listed in the report. The following changes were made in the manuscript to clarify this point.*

*Page 8, Lines 7-11:* *GMFCS level was reported to define to functional level for each individual in the CP cohort. In order to address changes in walking ability, the Functional Mobility Scale (FMS) was assessed. Both GMFCS level and FMS were scored by the physiotherapist conducting the gait analysis and were extracted from the gain analysis report.*

*We did not anticipate any changes in the GMFCS score as it was used as a functional characteristic and not as an outcome measure. Therefore, we have removed it from the functional variables. Thank you for bringing this to our attention.*

2. Page 6 – line 7-8: Human body model-2: Suggestion to use the reference of vd. Bogert et al., 2013 for HBM or Falisse et al., 2018 / Flux et al., 2020 for description of the updates in the updated version.

*Thank you for highlighting this. It has been updated in the manuscript.*

3. Line 17: Did the children truly walk barefoot on the treadmill? Or were non-supporting flexible shoes supplied as commonly used on the treadmill for safety and comfort?

*Our regular gait analysis procedure involves walking barefoot on the treadmill. In over 700 gait analyses, no patients reported discomfort or experienced issues pertaining to safety. As such, our experience suggests that walking barefoot is comfortable and safe, and does not appear to influence the results. This has been added to the manuscript:*

*Page 9, Lines 8-10: Individuals that are regular users of ankle-foot orthoses were usually assessed both barefoot and with their orthoses. However, for this study, only data from the barefoot condition were included.*

*Page 10, Line 2-3: No adverse events were reported during the assessments and none of the participants complained of discomfort when walking barefoot on the instrumented treadmill.*

4. Lines 23-13: Removing the signal of peroneus longus from analysis. Not sure why cross-talk of the peroneus would be a problem when conducting synergy analysis. Isn’t synergy analysis about activated groups of muscles together, instead of evaluating the pattern of individual muscles?

*Our experience led us to believe that due to the small size of the peroneus longus muscle and the close proximity to the neighboring muscles, the signal of the peroneous longus was often very inconsistent, to the extent that we were not confident that we were only measuring this muscle. As such, this would be a problem for including in the synergy analysis, as it could introduce an uncertain level of variability, potentially influencing the walk-DMC score. This has been expanded upon in the manuscript to provide more clarity*

*Page 8, Line 23- page 9, line 3: The data from the peroneus longus muscle presented very inconsistent activations patterns, possibly as a consequence of the small size of this muscle and its close proximity to the neighboring muscles (inter-muscular cross talk). The inclusion of this muscle in the synergy analysis could introduce an uncertain level of variability, potentially influencing the data. As such, this muscle was excluded from the study.*

5. Lines 19-20: Was the treadmill used in a self-paced mode or fixed?

*Due to difficulty with adapting to the self-paced mode on a treadmill, especially for those individuals who are unfamiliar with it, a fixed speed was applied to improve consistency between individuals. This has been clarified in the manuscript*

*Page 9, Line 12-13: All participants walked at a self-selected comfortable fixed speed.*

6. Line 21-22: Sufficient adaptation to walking on a treadmill: Please rephrase, as the term ‘sufficient’ is vague. It questionable whether 6 minutes are truly ‘sufficient’ in this population, as the study by Meyer 2019 was conducted on healthy adults and did not include EMG measurements.

*Thank you for highlighting this point. The word sufficient was removed:*

Page 9, Lines 12-16: *All participants walked at a self-selected comfortable fixed speed, determined according to feedback from the participants and their families, along with a minimum of six minutes walking to provide a period of* *familiarisation to walking on a treadmill.*

7. Page 7 – lines 3-5: Was the same walking speed imposed during the second assessment?
*No. Each participant defined their own comfortable self-selected walking speed in each of the assessments. This has been clarified in the manuscript.*

Page 9, Lines 24- page 10, line 2*:* *Both the first and second assessment protocols were identical, including the use of handrails by those participants who needed to do so. The only variable that changed was if the participant chose to walk at a different comfortable self-selected walking speed.*

Data analysis

1. Page 7 – lines 11-14: Was the visual quality check for EMG data done simultaneously for kinematics as described on page 8, lines 22-24? In other words, were the same strides included for these analyses? Or could this differ?

*Thank you for the comment. We have attempted to clarify these two points:*

Page 11, Lines 23-page 12 line 1*: Before calculating the GPS and GVS values, experienced clinicians performed a quality and consistency check of the kinematic data in the software Gait Offline Analysis Tool 4.2 (Motek, the Netherlands) to remove poor quality strides.*

Page 13, Lines 3-4*: the selected strides for extracting the walk-DMC and GPS/GVS were not always identical*

2. Page 9 – line 3: I assume the authors mean ‘the minimal clinically important difference’, instead of ‘mean important change’. The rest of this sentence is also unclear, please rephrase.

*Thank you for your comment. According to your previous comment regarding the GVS being calculated in this study by a different marker model (HBM2 instead of PiG used in the referenced article), the mean important change for the GVS values were deleted.*

3. Page 9 – lines 8-14: I don’t understand what is described in this paragraph? Which leg was used for analysis? It is unclear what is meant by ‘For the walk DMC, this was based on the less-involved leg to provide interparticipant consistency’. Please rephrase.

*Thank you for your comment. This was rephrased.*

Page 12, Lines 11-20: *When analyzing muscle synergies and kinematics for both legs together (bilateral analyses), the foot strike events of the right leg were used to split the signals into gait cycles for both sides (34).* *Based on preliminary testing on a subgroup of the participants, there was little influence on the bilateral walk-DMC value when segmenting the gait trials according to either the more or less involved leg. For the CP cohort, when muscle synergies and kinematics were extracted for the more involved leg, the foot strike events of that leg (the most involved) was used to split the signals into gait cycles. More or less involved leg was determined according to the clinical physical examination conducted as part of the clinical gait analysis protocol used in our lab.*

4. Page 9 – line 16: Typo, abbreviation dimensionless walking speed should be DWS.
*Thank you for notice. It was changed.*

Results
1. Page 10-11 , Table 1: Please indicate in the title that this table presents characteristics of participants with CP (not patients).

*Thank you for the comment. According to the editor and the second reviewer comments, we add the TD group into Table 1. Therefore, the title remained the same as the table describe both groups.*

2. Table 1: Can the authors provide information on the type of orthopaedic interventions? As a percentage of 49% is quite high. Furthermore, it should be explained more clearly what the numbers in the table represent. Please indicate per outcome whether it concerns a number, mean, SD or range (min-max). The age range in the Table does not match the range 4-18 described on page 5.

*Information regarding previous orthopedic interventions were added to table 1. Additionally, the age range was deleted from page 5.*

3. Page 11: Longitudinal changes in SMCg: Were data normally distributed? I.e. do the results represent outcomes of parametric or non-parametric tests?

*This depends on the variable. The data that was normally distributed was analyzed using parametric statistics, and the data that was not normally distributed was analyzed using non-parametric statistics. We added it to the manuscript.*

Page 14, Lines 19-20*: Walk-DMC scores for the more involved leg and for both legs together, from both assessments, were normally distributed, and are presented in Table 2. And-* Page 16, Lines 6-7*: The GPS values for the more involved leg and both legs together, in both assessments, were not normally distributed, and are presented in Table 2.*

4. Again, splitting outcomes in ‘on group level’ and ‘on the individual level’ is confusing, as the used statistics should account for inter-individual differences. The authors found no statically significant changes in SMCg. The fact that there are large inter-individual differences is nice to present as additional information, but it should not be presented as ‘changes on individual level’.

*Thank you for your comment. According to your suggestion, we deleted the term "on the individual level".*

5. Page 11-12 - Table 2: GPS value- total value also refers to both legs together?

*Yes, this has been rephrased and added to Table 2*.

6. Page 12 – line 2: Personally, I would not expect any change in GMFCS, even if there would be a change in functional level as this outcome is probably not sensitive enough to detect these changes. Actually, it is meant to be robust against (natural/expected) functional changes during growth and development. Reconsider using this as an outcome of ‘change on a functional level’.

*Thank you for bringing this to our attention. As addressed in one of your earlier questions, the GMFCS level has been removed as a functional variable, and remains as a descriptive variable for the individuals in the CP cohort.*

7. Page 13 – line 1-2: Why are no results presented of the MAP-score for both assessments in Figure 2? This is required to place the findings of the hip GVS in perspective. At this point, I sincerely doubt whether the significant relation between the change in hip joint GVS and walk-DMC is not just a lucky shot.. Results showing mean +/-SD for both assessments should be added.

*In* Page 16, Lines 11-12 *it was stated that "* *no significant change was found in the median GPS or GVS values between the first and second assessment". As such, the mean and SD were not included (as is the experience of the authors in previous publications e.g., 10.1016/j.rehab.2022.101652). However, as requested, we have added the mean together with the 95% CI line for each bar in figure 2. We hope this provides you with more clarity for interpreting the MAP figure.*

8. Page 13 – lines 1-2: Using the terms GVS and MAP alternately throughout the paper is confusing, especially for readers who are less familiar with these concepts. It would benefit readability to choose one of them.

*Thank you for your comment. We have used the MAP only where there is a description of all the GVS together and for figure 2's description (as specified in the paper of Baker et al 2009). The term GVS was used in the manuscript according to your suggestion.*

9. I have a comparable issue with the terms SMCg and DMC, as both abbreviations basically represent the same phenomena. Sticking to one term makes it a lot easier to read.

*Thank you for your comment. According to your suggestion, we have used the term SMCg in the manuscript. DMC was only meant to relate to the outcome measure walk-DMC.*

10. Page 13 – line 15 to page 14 line 5: Remark on using the MCID values for GVS on the current dataset. The values of 1.5 deg and 0.6 deg for the hip joint reported by Baker et al are based on the PlugInGait model. However, the underlying biomechanical calculations for HBM are different, using global optimisation to model markers. This may especially affect hip rotation, which is measured with just one lateral marker on the thigh.
*Thank you for bringing this to our attention. This changes in GVS based on the MCID of Baker et al. (2012) have been removed, and are now presented in a figure (figure 4).*

Page 17, Lines 12-22*:* *However, with respect to the GVS, a significant moderate negative correlation was found between the change in walk-DMC to the change in hip flexion/extension GVS value in the more involved leg (r_p_=-0.56, p=0.017; Fig 3a). These findings indicate that when the walk-DMC value increased (improved SMCg complexity), there was less deviation in hip flexion/extension kinematics. Moreover, a significant moderate positive correlation was also found between the change in walk-DMC and the hip internal/external rotation GVS value in the more involved leg (r_p_=0.65, p=0.004, Fig 3b). These findings indicate that when the walk-DMC increased, (improved SMCg complexity), there was more deviation in hip internal/external kinematics. An overview of the changes in the hip flexion/extension and hip internal/external rotation GVSs between the assessments is presented in Fig 4.*

Discussion
1. Page 14 – line 9: ‘confirming the first hypothesis’. What was the first hypothesis? Please repeat the hypothesis or rephrase this sentence (same for the second hypothesis in line 12).
*Thank you for your comment. We rephrased it*

Page 18 Lines 10-16*: This study evaluated longitudinal changes in SMCg in individuals with CP, and whether this related to changes in gait deviations. There was no change in walk-DMC between assessments, confirming the first hypothesis. However, high variability was found between participants for the more involved leg. The change in Walk-DMC score was found to relate to changes in hip flexion-extension and internal-external rotation GVSs between assessments. This partially confirmed the second hypothesis that changes in SMCg will relate to changes in gait deviations.*

2. Line 9: As mentioned above. Please drop the words ‘on individual level’.

*According to your suggestion we deleted the term "on the individual level".*

3. Page 14 – lines 10-11: I assume the authors mean that the change in walk-DMC was related to changes in hip flexion – extension … GVS? As mentioned above, consider to use just one term for reporting the outcomes of motor control.

*This has been clarified in the manuscript.*

Page 18, Lines 13-15*: The change in Walk-DMC score was found to relate to changes in hip flexion-extension and internal-external rotation GVSs between assessments.*

4. Page 15 – lines 1-2: A conclusion of this paragraph is missing. What is the point of repeating the finding that the authors found no change in SMCg? What is their interpretation?

*Thank you. We have attempted to address this by improving the clarity of the first paragraph, and moving the final sentence to the beginning of the next paragraph:*

*Page 19, Lines 1-8:* *Prior to evaluating changes in SMCg over time, we explored the relationship between age and walk-DMC at the time of the first assessment, without any significant findings. This is in line with a previous study in a very large cohort (12). Additionally, Cappellini et al also demonstrated very limited age-related changes in muscle synergies in children with CP, based on a single assessment in a wide age range of participants (13). It has also been shown that SMCg in individuals with CP can often resemble the synergies observed in much younger TD children, highlighting not only the delay but also stagnation in SMCg development (40).*

5. Lines 3-9: As described above. I continue to have a problem with reporting this finding as improvement on an individual level. In my opinion, it could also mean that (1) there is no change over time (2) the variability could also raise questions on the robustness of this outcome.

*According to your suggestion we deleted the term "on the individual level". We have also rephrased the discussion with respect to your concerns (see changes made in the responses to this pint in previous questions).*

6. Lines 10-13: See above on my questions regarding these outcomes of the hip joint. More extensive presentation of the results is required to support this conclusion.

*Thank you for your comment. We have addressed this with the addition of a figure (figure 4).*

7. Lines 13-17: This is quite speculative and should rephrased. See previous comment.

*Thank you for your comment. We rephrased it*

*Page 20, Lines 3-13: In a previous study, it has been suggested that level of SMCg might be indicative of the capacity of the central nervous system to selectively activate muscles during a dynamic task. This capacity might also affect gait kinematics, with more alterations across multiple joints when SMCg is more impaired (21). However, a direct association between a gait pattern and level of SMCg was not found in this study, similar to our results for the GPS. However, we did find that the sagittal and transverse plane hip kinematics might be associated with level of SMCg. The importance of sagittal hip position in regulating motor control of walking in cats was reported over 20 years ago (41). However, whether hip position actually plays an important role in regulating human walking or what the mechanism behind this association may be, remains undetermined.*

8. Lines 22-24: Could habituation to treadmill walking also affect these result, leading to a more upright posture during the second assessment?

*Thank you for your comment. Since we did not document treadmill experience before the first assessment or between the first and second assessments, we are unable to support your suggestion with data. However, subjectively, we are aware that most of the individuals in this study are familiar with treadmill walking during their routine physical therapy treatments. This has been addressed in the methods*

Page 10, Lines 5-8*:* *Moreover, it should be noted that the participants continued to receive their usual clinical treatment between assessments, including physical therapy (often with gait training that may involve treadmill walking), occupational therapy, hydrotherapy, and other relevant non-surgical interventions.*

How many children used the handrail for support? And did this change between assessments?

*Most participants (n=19) did not use handrails. The initial analysis did not reveal any significant influence of handrail use on the results. It should also be noted that these participants used handrails in both assessments, which has now been clarified in the manuscript*

*Page 9, Line 24-25:* *Both the first and second assessment protocols were identical, including the use of handrails by those participants who needed to do so.*

*Page 14, Line 14-16:* *Most participants (n=19) did not use handrails during the assessments; an initial analysis did not reveal any significant effect of using handrails on the results.*

9. Page 16 – lines 4-5: This is no causal effect, as muscle co-contraction and motor control quantified by muscle synergies capture different phenomena. Please rephrase.

*Thank you. We wrote muscle co-activation and not co-contraction (as you wrote in your question), with the former being more generic in definition. We consider the muscle synergy analysis purely to be a measure of muscle co-activation during a particular task, where the findings (i.e. walk-DMC or tVAF) are often inferred to be related to motor control. As such, we are confident in our statement that less lower limb muscle co-activation during gait will lead to a better walk-DMC score. However, in an attempt to be as concise as possible, we have made the following modification:*

*Page 21 lines 1-6:* *An increase in hip extension during the gait cycle can result in a more extended posture and posterior shift of the center of mass, optimising the alignment of the ground reaction force around the hip, knee and ankle (42). This could alter the activation timing of the hamstring muscles (as hip extensors) and quadriceps muscles (as knee extensors) during the stance phase, resulting in less lower limb muscle co-activation, and, a better walk-DMC score.*

10. Page 16 – lines 7- to end: Again, I have doubts on the importance of the results in the hip joint. Especially for quantification of hip internal-external rotation using the HBM model. This paragraph of the discussion is speculative and puts (in my opinion) too much emphasis on the results of the hip rotation. In contrast, a critical discussion of the findings of this paper and the limitations of the methods used is missing at this point.

*In order to address you concerns, we have removed some of the potential explanations for the change in hip rotation, and strengthened the wording of the need for further study to determine the clinical relevance.*

Page 21, Lines 14-21: *Regarding the transverse plane hip ROM, a change in hip internal/external rotation was correlated with a change in walk-DMC. Further analysis revealed that unlike hip flexion/extension, the direction of the change was highly variable between participants. This may be a consequence of changes in femoral anteversion and instability around the foot, two clinical findings that influence the lever arm of the external hip rotator muscles (41,42) and may influence muscle activity and SMCg. Further study is required to determine the clinical relevance of the finding of changes in hip internal/external rotation GVS with walk-DMC.*

11. Page 18 – lines 6-11: Including different strides for kinematics and EMG. This should be included in the results sections or methods. Moreover, it is not clear what point the authors try to make by saying that they therefore ‘did not evaluate their interaction’.

*Thank you for the comment. The word interaction was removed, and this point is introduced in the methods instead of discussion.*

*Page 12, Line 3-9: The selected strides for extracting the walk-DMC and GPS/GVS were not always identical. For example, there were strides that contained good quality EMG data, but not kinematic data. These strides were only used for extraction of the walk-DMC, and not for the calculation of the GPS or GVS. However, due to the large number of strides that were included it is not anticipated that this would lead to a change in the results.*

12. Lines 14-16: Is it known whether participants walked on the GRAIL or another treadmill system during the interim period in-between assessments? See my question about habituation. Were children seen by other therapists or did they have any other therapy or training that may affect their gait

*Thank you for your comment. This has been answered in your question 8.*
